# Propranolol reinstates mitochondrial dynamics and synaptic memory pathways through CaMKII/CREB–BDNF/ PKMζ cascades in an AD-like rat model

**DOI:** 10.3389/fnagi.2026.1729046

**Published:** 2026-05-29

**Authors:** Pinky, Neha, Medha Kaushik, Prachi Tiwari, Mohamed El-Tanani, Syed Arman Rabbani, Suhel Parvez

**Affiliations:** 1 Department of Toxicology, School of Chemical and Life Sciences, Jamia Hamdard, New Delhi, India; 2 Department of Paramedical Sciences, School of Rehabilitation Health Sciences, Jamia Hamdard, New Delhi, India; 3 RAK College of Pharmacy, RAK Medical and Health Sciences University, Ras Al Khaimah, United Arab Emirates; 4 Department of Clinical Pharmacy and Pharmacology, RAK College of Pharmacy, RAK Medical and Health Sciences University, Ras Al Khaimah, United Arab Emirates

**Keywords:** Alzheimer’s disease, propranolol, mitochondrial dynamics, synaptic plasticity, CaMKII/CREB signaling, BDNF pathway, PKMζ

## Abstract

**Background:**

Alzheimer’s disease (AD) is a major neurodegenerative disorder characterized by amyloid-β (Aβ) accumulation, neurofibrillary tangles, and progressive cognitive decline. Despite significant advances in understanding its pathophysiology, current therapeutic options provide limited symptomatic relief. The present study investigated the nootropic and anti-amnesic effects of propranolol (PRO) in a scopolamine (SCP)-induced AD-like rat model.

**Methods:**

Wistar rats received PRO (10, 30, or 50 mg/kg, p.o.) or donepezil (DPZ; 1 mg/kg) for 17 days. Cognitive deficits were induced by SCP (1 mg/kg, i.p.) administration from day 9 onward. Behavioral performance was assessed using the Novel Object Recognition (NOR) and Elevated Plus Maze (EPM) tests. Molecular and cellular analyses were conducted to evaluate synaptic plasticity markers (CaMKII, CREB, BDNF, PKMζ), mitochondrial function, oxidative stress parameters, and inflammatory markers (GFAP, TNF-α).

**Results:**

Propranolol treatment significantly improved long-term memory performance, enhanced recognition index, and attenuated anxiety-like behavior in SCP-treated rats. These behavioral effects were associated with upregulation of CaMKII–CREB–BDNF–PKMζ signaling, improvement in mitochondrial membrane potential (Δψm), reduction in reactive oxygen species (ROS) generation and Aβ1–42 accumulation, and decreased expression of GFAP and TNF-α.

**Conclusion:**

The findings suggest that propranolol mitigates SCP-induced cognitive impairments, potentially through modulation of synaptic plasticity– related signaling, mitochondrial function, and neuroinflammatory responses. These results indicate the therapeutic potential of propranolol in experimental models of AD-related neurodegeneration, warranting further investigation.

## Graphical Abstract

**Figure:**
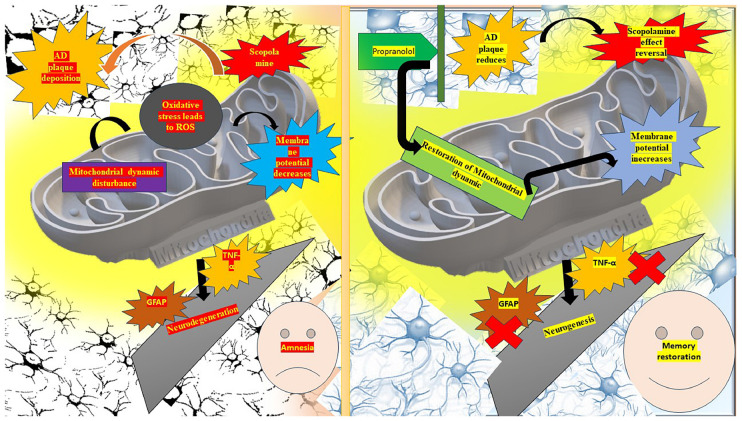
Sketchmatic representation of the study: SCP-induced toxicity vs. PRO memory restoration.

## 1 Introduction

Memory loss appears to be one of the earliest symptoms of AD that patients and their caregivers report experiencing. Early in the course of the disease, both working memory and long-term declarative memory are compromised (Pinky et al., 2023a). Most people agree that plasticity is essential to learning and memory. Many signaling pathways, including several protein kinases and phosphates, are necessary for the formation of long-term memory (Nguyen and Woo, 2003). Numerous *in vitro* and *in vivo* investigation have demonstrated the critical function of several proteins and complexes in promoting memory consolidation within the prefrontal cortex and other brain areas. CaMKII is thought to be the primary modulator of synaptic plasticity, exerting significant influence over the brain’s learning and memory functions. CaMKII signaling is not only involved in memory formation but is also impaired in the brains of AD patients (Yasuda et al., 2022). It has been discovered that Aβ_1–42_ toxicity increases intracellular calcium concentration in cultured neurons, which results in cell death. Furthermore, transgenic AD mice exhibit elevated calmodulin (CaM) expression and decreased p-CaMKII (Min et al., 2013). It is well recognized that CREB is essential to the basic processes of memory and learning as well as to additional complicated brain activities including addiction, anxiety, and depression (Crews and Masliah, 2010). It has also been discovered that activated CREB promotes BDNF production, a crucial biochemical component for memory and cognitive preservation. According to Shivarama Shetty and Sajikumar (2017), long-term memory consolidation requires the upregulation of CREB-BDNF signaling. Long-term memory maintenance is significantly influenced by the brain-specific, atypical protein kinase C isoform known as protein kinase Mζ (PKMζ). This molecule is necessary for a variety of learning modalities, including fear conditioning and spatial memory, as well as long-term neuronal potentiation (Chan et al., 2016).

A number of research have determined that mitochondrial dysfunction plays a significant role in the etiology of AD (Pinky et al., 2023b). Aβ oligomer insertion into the bilayer may cause ROS to be produced, which would then start the peroxidation of membrane lipids intercellular proteins, and nucleic acids (Allan Butterfield and Boyd-Kimball, 2018). Reduced ROS may lower the development of AD by shielding brain mitochondria from oxidative damage. Oxidative stress happens when the body cannot keep the production and consumption of oxidant molecules in balance. The formation and progression of AD are also significantly influenced by inflammation, and a variety of cytokines, such as TNF-α is important in the pathogenesis of AD. Furthermore, SCP-induced dementia has been widely employed to evaluate possible therapeutic drugs for the treatment of AD. A nonselective muscarinic cholinergic receptor antagonist, scopolamine is linked to cholinergic dysfunction, which results in impairments in memory and learning (Falsafi et al., 2012).

Often used to treat acute anxiety, angina pectoris, cardiac arrhythmia, and hypertension, PRO is a β-adrenergic antagonist. Research has demonstrated that PRO can cure stress-related cognitive impairments (Shankle et al., 1995; Wang et al., 2007) and lower Aβ levels *in vitro* (Dobarro et al., 2013). In the past, PRO has been employed as a therapeutic drug to address disruptive behavior in AD, primarily in agitation and aggression. In the Tg2576 transgenic mice model of AD, PRO is suggested to potentially improve memory deficits and Aβ pathology at doses lower than those used as antihypertensives (Wang et al., 2007). While these findings were obtained in transgenic mouse models of Alzheimer’s disease, the present study employs a pharmacological scopolamine-induced amnestic model in adult Wistar rats to examine propranolol’s effects on memory, synaptic plasticity, and mitochondrial signaling under AD-like conditions. Propranolol is a non-selective β-adrenergic receptor antagonist with high lipophilicity, a property that enables efficient penetration across the blood–brain barrier. Unlike peripherally restricted β-blockers, propranolol readily accumulates in brain tissue and directly modulates central noradrenergic signaling. In the central nervous system, β-adrenergic receptors regulate synaptic plasticity, memory consolidation, stress responsiveness, and neuronal excitability through cAMP-dependent signaling pathways. Dysregulated β-adrenergic signaling has been implicated in cognitive impairment, neuroinflammation, and mitochondrial dysfunction, all of which are key contributors to Alzheimer’s disease–related pathology. By antagonizing excessive β-adrenergic activation, propranolol is proposed to normalize aberrant neuronal signaling, reduce stress-induced synaptic disruption, and indirectly influence downstream plasticity-related pathways such as CaMKII, CREB–BDNF, and PKMζ.

Even though PRO has various uses, there have been no neuropharmacological studies for dose comparison of PRO on memory deficits in the prefrontal cortex of a rat model of cognitive impairment caused by SCP. This study first investigated the effectiveness of different doses of propranolol (10 mg/kg, 30 mg/kg, and 50 mg/kg) and also compared them with the clinically used FDA-approved drug DPZ. Furthermore, no research has been done to examine the possible synaptic plasticity-related markers CaMKII, CERB-BDNF, and PKMζ for memory restoration to understand how this medication causes an anti-amnesia effect. It is important to note that the scopolamine model represents an acute, reversible cholinergic dysfunction and does not fully recapitulate the progressive neuropathological features of Alzheimer’s disease; therefore, it is best interpreted as an AD-like amnestic model suitable for probing memory-related mechanisms rather than disease progression.

## 2 Materials and methods

### 2.1 Ethics and animal care

The National Institutes of Health (NIH publication no. 85–23, amended 1996) and ARRIVE criteria for animal care and use were adhered to in the experimental methodology. The central animal house facility of Jamia Hamdard, New Delhi, India, provided adult male Wistar rats aged 8–10 weeks, weighing between 220 and 250 g. They were housed at 22 °C ± 3 °C with ambient temperature, 47%–55% relative humidity, and 12 h of light and dark cycles. The animals were fed a conventional diet of rat pellets and filtered water *ad libitum*. Every experiment was carried out according to the Committee for Control and Supervision of Experiments with Animals (CPCSEA) rules. The Institutional Animal Ethics Committee (IAEC) of Jamia Hamdard (Registration No. 173/GO/ReBi/S/2000/CPCSEA) approved the experimental protocol.

### 2.2 Drugs and chemicals

The chemicals, reagents, proteins, and ligands were procured from various manufacturers. Scopolamine (1610001), Propranolol Hydrochloride (P0884), Donepezil (D6821), DCFDA, and RIPA (9806S) (Sigma-Aldrich, St. Louis, MO, USA) Rhodamine123 (Rh123) was obtained from (cat no. 2313036; Invitrogen), Ethanol, Tween 20, Methanol, PBS, Trizma, Glycine, Bovine serum albumin (BSA), Ketamine, Xylazine, RIPA lysis buffer (CST; 9806S), and a protease inhibitor cocktail (Abkine, BMP1001), which were purchased from Hi-Media and Merck labs Pvt. Ltd., Mumbai, India.

### 2.3 Experiment design

**FIGURE 1:**
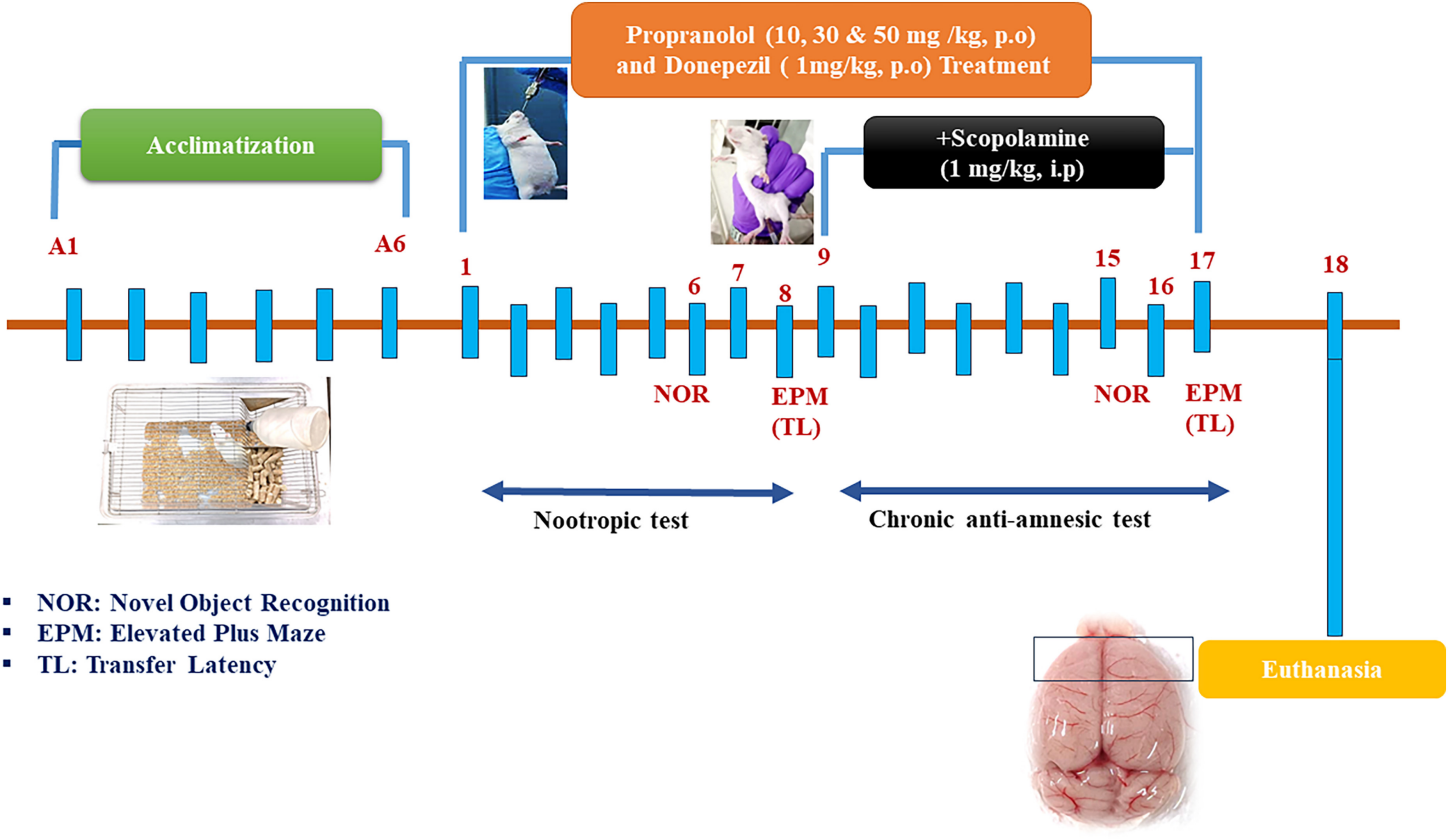
Schematic representation of the experimental timeline and procedure. IAEC approved the animal testing studies, and CAHF procured male wistar rats aged (220–250 g). The experiments comprised of two parts: Experiment 1 (nootropic model) involved oral administration of PRO or DPZ without SCP exposure to evaluate intrinsic effects on learning and memory, followed by NOR and EPM testing on days 6–8. Experiment 2 (SCP-induced amnestic model) involved oral pretreatment with PRO or DPZ for 17 days, with SCP (1 mg/kg, i.p.) administered from days 9–17 to induce cognitive impairment. Behavioral testing was conducted during scopolamine exposure, and animals were euthanized on day 18 for molecular, biochemical, and histological analyses.

The experimental design comprised two distinct but complementary paradigms: (i) a nootropic model to assess the intrinsic effects of propranolol on learning and memory in healthy rats, and (ii) a scopolamine-induced amnestic model to evaluate the protective and restorative effects of propranolol against cholinergic memory impairment. These two paradigms were conducted independently using separate cohorts of animals and differed in treatment duration, scopolamine exposure, and behavioral testing timelines, as detailed below and schematically illustrated in Figure 1.

#### 2.3.1 Drug treatment

For the first time, the three-dose ranges of PRO doses were taken into consideration for the comparative evaluation. Specifically, low (10 mg/kg), medium (30 mg/kg), and high (50 mg/kg) doses were selected to capture a graded spectrum of central β-adrenergic modulation and to enable assessment of dose-dependent behavioral and molecular effects. The range of the doses for PRO was determined based on a review of the literature or previous studies (Kroon and Carobrez, 2009; Dobarro et al., 2013; Xue et al., 2017). These studies have demonstrated that propranolol within this dose range effectively crosses the blood–brain barrier and produces measurable central nervous system effects without inducing overt systemic toxicity. PRO was solubilized in distilled water (dw). DPZ and SCP were prepared in dw as well. Throughout the trial, distilled water was given to normal control rats. In the case of PRO and DPZ, the medication was administered orally, while SCP was administered intraperitoneally (i.p.). To prevent order and time from having an impact, all tests were carried out using a balanced design with nine animals. The nootropic and SCP models were the two categories into which the behavior experiments were divided (Kroon and Carobrez, 2009).

Nootropic Model

G1: control (dw); *n* = 9
G2: positive control DPZ (1 mg/kg, p.o); *n* = 9
G3: Low dose of PRO (10 mg/kg); *n* = 9
G4: Medium dose of PRO (30 mg/kg); *n* = 9
G5: High dose of PRO (50 mg/kg); *n* = 9

All groups received oral pretreatment for the nootropic action of above above-listed drugs. From day six to day eight, all of these rats underwent behavior testing for NOR and EPM (Figure 1).

Scopolamine Model

G1: control (dw); *n* = 9
G2: negative control SCP (1 mg/kg, i.p.); *n* = 9
G3: positive control DPZ (1 mg/kg, p.o); *n* = 9
G4: Low dose of PRO (10 mg/kg, p.o); *n* = 9
G5: Medium dose of PRO (30 mg/kg, p.o); *n* = 9
G6: High dose of PRO (50 mg/kg, p.o); *n* = 9

All animals in the SCP model, except the control group, received daily intraperitoneal injections of scopolamine (SCP; 1 mg/kg) from day 9 to day 17 to induce amnesia. PRO or DPZ was administered orally once daily for a total duration of 17 days, with SCP administration beginning after the initial 8 days of drug pretreatment. Behavioral assessments were conducted during ongoing SCP exposure, with the Novel Object Recognition (NOR) test performed on days 15–16 and the Elevated Plus Maze (EPM) on day 17, 1 h after scopolamine injection. Following completion of the behavioral assessments on day 17, animals were deeply anesthetized with ketamine (75 mg/kg, i.p.) and xylazine (10 mg/kg, i.p.). Adequate depth of anesthesia was confirmed by the absence of the pedal withdrawal reflex. Euthanasia was then performed via transcardial perfusion under deep anesthesia as the terminal procedure. Death was confirmed by cessation of heartbeat and respiration prior to tissue collection. Brains were immediately excised, and the prefrontal cortex was rapidly dissected for subsequent molecular, biochemical, and histopathological analyses. All procedures were carried out in accordance with the guidelines of the National Institutes of Health (NIH) and the Committee for the Purpose of Control and Supervision of Experiments on Animals (CPCSEA).

### 2.4 Neurobehavior

#### 2.4.1 Long-term memory test

A well-known neurobehavioral activity for assessing rats’ recognition memory is Novel Object Recognition (NOR). With a few minor adjustments, the test was carried out by the methodology (Kroon and Carobrez, 2009). The three phases of NOR assessment were (i) habituation, (ii) familiarization, and (iii) the test phase. The NOR apparatus was a rectangular chamber with dimension of 50 cm × 50 cm × 35 cm. the apparatus was well equipped with a digital camera placed at the top of the chamber, providing an overhead view. All neurobehavioral observations were recoded using ANY-maze v6.2 (Stoelting Co.) connected to the apparatus. Rats were habituated to the apparatus for 5 min without any object the day before the trial. Trial 1 on the next day involved exposing each rat to two known objects for 5 min. Similarly, the two trials were conducted. The next day, after the trials had been completed, rats were exposed to the test phase, where a novel object (NO), a new object, took the place of one familiar one. To reduce bias and mistakes, the NO was always positioned in the same location as the preceding object. ANY-Maze software was used to record and assess the amount of time spent with each object. To reduce scent trails, the open-field box was cleaned with 70% ethanol in between runs. The formula [TB/(TA + TB) ×100] was used to determine the recognition index percentage, where TA and TB represent the amount of time spent studying known item A and novel object B, respectively (Kroon and Carobrez, 2009). When a rat sniffed or touched an object, it was considered to have been explored.

#### 2.4.2 Anxiety behavior test

A common method for identifying rodents’ anti-anxiety behavior is the elevated plus maze (EPM). The EPM test equipment was made up of two open arms that spanned over two closed arms with high walls, or four arms with the same dimensions. Because a central square was used to link these arms, the contraption took on the appearance of a plus sign. In addition, the EPM was raised above the floor (Bhuvanendran et al., 2018). Dim red light lighting was used during the day to conduct the behavior tests. Two sessions were dedicated to the EPM assessment of memory. Every rat’s latency time (s), or the amount of time it took for it to enter (completely pawed) into a closed arm, was recorded during the training phase. To reduce scent traces, the maze was cleaned in between runs using 70% ethanol. A test phase was held 24 h (retention) following a training session to assess memory retention. Each rat had 90 s to explore the maze during the training and test periods. Throughout the session, a drop-in transfer latency time was used as an indicator for memory enhancement.

### 2.5 Tissue processing and mitochondria isolation

#### 2.5.1 Western blotting (whole cell lysate)

Rats were immediately sacrificed for brain excision on test day following the EPM test. For western blot analysis, PFC tissue from both hemisphere of the brain was obtained, snap-frozen, and kept at 80 °C. In summary, lysis buffer (10% RIPA in nuclease free water with 1 % protease inhibitor cocktail) was used to prepare the tissue homogenate. The blended material underwent sonication and centrifugation. After that, the supernatant was collected, and the Bradford assay was used to quantify the protein. Using a Mini Trans-Blot Cell device (Bio-Rad), samples were resolved using a 10%–12% SDS-PAGE gel at 20 mA. Afterward, they were transferred to PVDF membranes and left for 1.5–2 h at 150 mA for two gels. Following an hour of blocking at room temperature with 5% non-fat skim milk (Sigma-Aldrich), each membrane was washed with PBST for 5 min. Following that, the membranes were incubated for an additional night at 4 °C with primary antibodies, such as the PKMζ antibody (1: 1000, Abcam, #ab59364) and the BDNF antibody (1:1000, Gentex #FTX132621). The following day, three PBSTs were washed, and membranes were treated with an anti-rabbit secondary antibody coupled with horseradish peroxidase (1:10,000, Invitrogen). Bands were visualized using an enhanced chemiluminescence reaction substrate (SuperKine TM West Pico Plus; Abkine), and densitometric analysis was performed using ImageJ software (Bellezza et al., 2018).

#### 2.5.2 Immunohistochemical analysis for Aβ, TNF-α, CaMKII, and CREB

Immunohistochemical staining was carried out following the protocol detailed in a previous study (Quadri et al., 2018). In summary, the animals were anesthetized and then perfused with cold, normal saline. Subsequently, they were fixed with a 4% paraformaldehyde solution, and tissue sections were sliced from the paraffin-embedded specimen using a microtome. After removing paraffin using xylene and dehydrating with varying concentrations of graded ethanol, antigen retrieval occurs through boiling in citrate buffer. Following the washing step, a blocking procedure was performed, and the sections were then subjected to incubation with the primary antibodies targeting Aβ (1:100, CST, #D3D2N), TNF-α (1:100, CST, #D2D4), CaMKII (1:100, CST, #3362S), and CREB (1:100, CST, #D76D11) overnight at 4 °C. Afterward, following the washing step, the samples were subjected to incubation with an IgG anti-rabbit secondary antibody (1:10,000, Abbkine, #A21020) and an IgG1 anti-mouse secondary antibody (1:10,000, Abbkine, #A21020). Subsequently, staining was carried out using 3,3-diaminobenzidine (DAB; Sigma Aldrich, USA) in butyl phthalate polystyrene xylene and examined using a fluorescence microscope at 20× magnification (Zeiss Microscopy; ZEN Blue Edition).

#### 2.5.3 Immunofluorescence analysis of GFAP

Immunofluorescence (IF), a crucial immunological technique, is employed to detect and pinpoint various antigens within different cell samples and tissues. In the case of brain tissue slides preserved in formalin and embedded in paraffin, the following steps were carried out for immunofluorescence: First, deparaffinization and hydration procedures were executed on 3 μm-thick paraffin sections. Subsequently, these sections underwent heat-induced antigen retrieval in a citrate buffer with a pH of 6.0. For hippocampal sections, Triton-X (0.3%) was applied and left to incubate for 30 min at room temperature in a blocking solution containing 1.5% (wt/vol) BSA (Quadri et al., 2018). The primary antibodies (GFAP, etc.) were diluted using phosphate buffer and then incubated overnight at 4 °C to remove unbound antibodies the sections were washed 3 times in 1× phosphate buffer. Subsequently, the DyLight 488 goat anti-rabbit secondary antibody (Abbkine, A23220) was applied to the sections after being diluted 1:200 in phosphate buffer and left to incubate for an hour at RT. The slides were mounted with DAPI which was prepared from stock solution of 10 mg/ml and diluted at the ratio of 1: 10,000 for nuclei counterstaining (Fluoroshield; GTX30920). After a buffer wash and mounting, the slides were examined under a Zeiss microscope at 20×. For immunofluorescence analysis, three independent animals per group were used, and three to five sections per animal were examined under identical imaging settings.

#### 2.5.4 Quantification of immunostaining images

ImageJ software (NH, Bethesda, MD, USA) was used to analyze the images of the immune-stained sections. Three to five anatomically matched sections per animal were analyzed, with three independent animals per group used for immunohistochemical quantification unless otherwise stated. For each section, three randomly selected, non-overlapping fields were quantified to minimize regional bias. We used an optical microscope (ZEN Blue edition) to observe the expression patterns of these targets, and we took pictures with a digital camera (Axiocam 503 color from Zeiss). The point count approach was used to evaluate the immunological reactivity’s volume density. Also, a grid-cycloid-arc pattern with a predetermined number of points was superimposed. The points, which are the grid’s vertical and horizontal line intersections, that fall on the positively stained regions were tallied. The volume density of the positively stained structure is given by the ratio of the counted points to the total number of points in the grid.

#### 2.5.5 Histological assessment

Ketamine (75 mg/kg) and xylazine (10 mg/kg) were used to anesthetize randomly selected rats (*n* = 3–6 per group). For each animal, three to five serial coronal sections of the prefrontal cortex were analyzed following H&E and cresyl violet staining. A 4% paraformaldehyde (PFA) perfusion was administered after they were put to sleep. With the use of H&E and CV staining, the rat PFC underwent histopathological analysis. The brain tissue was fixed in 4% PFA overnight and embedded in paraffin for 24 h. The samples were washed, dehydrated by alcohol, cleared in xylene, and embedded in paraffin at 56 *^o^*C in a hot air oven for another 24 h. A 5-μm-thick PFC brain section was cut and put on glass slides for staining. H&E and CV dye were used to stain the sections. Images were taken with a Zeiss microscope (ZEN Blue edition) at a 20× magnification (Mandour et al., 2021).

### 2.6 Flow cytometry analysis

For all experimental analyses, biological replicates refer to independent animals within each treatment group. Behavioral experiments were conducted using nine animals per group. For molecular and biochemical analyses, subsets of animals were used as specified below. Western blotting analyses were performed using tissue from three independent animals per group, with each sample processed and analyzed independently. Flow cytometry–based mitochondrial assays were conducted using mitochondria isolated from two to four independent animals per group, as indicated in the respective figure legends. For histological, immunohistochemical, and immunofluorescence analyses, three to ten animals per group were used depending on the assay, and multiple sections were analyzed per animal to ensure reproducibility. Technical repeatability was ensured by performing identical staining, imaging, and quantification procedures across all groups under blinded conditions.

#### 2.6.1 Estimation of mitochondrial ROS production

The development of multiple neurodegenerative disorders, such as AD has been linked to oxidative stress and mitochondrial damage. In the present study, flow cytometry techniques were used to analyze the ROS using dichloro-di-hydro-fluorescein-diacetate (DCFDA) (Salman et al., 2020). Isolated mitochondrial fractions were incubated with 10 μM DCFDA dye for 5 min in the dark at room temperature (Mailloux, 2021). The FACS acquisition of MFI from 10,000 events was done using BD-LSRII (BD Sciences).

#### 2.6.2 Assessment of mitochondrial membrane potential (Δψm)

Oxidative stress can lead to the overproduction of ROS, which can lead to mitochondrial DNA mutations, harm the respiratory chain in the mitochondria, change membrane permeability, and affect Ca^2+^ homeostasis and the mitochondrial defense mechanisms. By causing or exacerbating neuronal dysfunction and instigating neurodegeneration, all of these alterations are linked to the onset of various neurodegenerative illnesses. In the present study, mitochondria were isolated by different centrifugation forces, and ▲ψm was measured by flow cytometry (Salman et al., 2020). Briefly, isolated mitochondria from PFC were diluted in an analysis buffer and incubated with 100 nM rhodamine 123 dye for 30 min at RT in the dark. The flow cytometry acquisition if MFI from 10,000 events was made using the BD-LSR II, and histogram were generated using the FACS-DIVA analysis software.

### 2.7 Statistical analysis

The present study signified the comparisons between SCP and treated groups of PRO and DPZ were evaluated using one-way analysis of variance (ANOVA), in continuation with Turkey’s multiple comparison and uncorrected Fisher’s LSD multiple comparison tests. A statistical study was supported using GraphPad Prism 8.0 version (GraphPad software, San Diego, CA, USA). Value was expressed as mean ± SEM and the nominal level of significance was identified at *p* < 0.05. All behavioral scoring, image acquisition, and quantitative analyses were performed by investigators blinded to the treatment groups to ensure repeatability and minimize experimental bias.

## 3 Results

### 3.1 PRO restores cognitive functions against SCP-induced cognitive deficits

#### 3.1.1 PRO administration induces long-term memory restoration by showing anti-anxiety behavior

**FIGURE 2:**
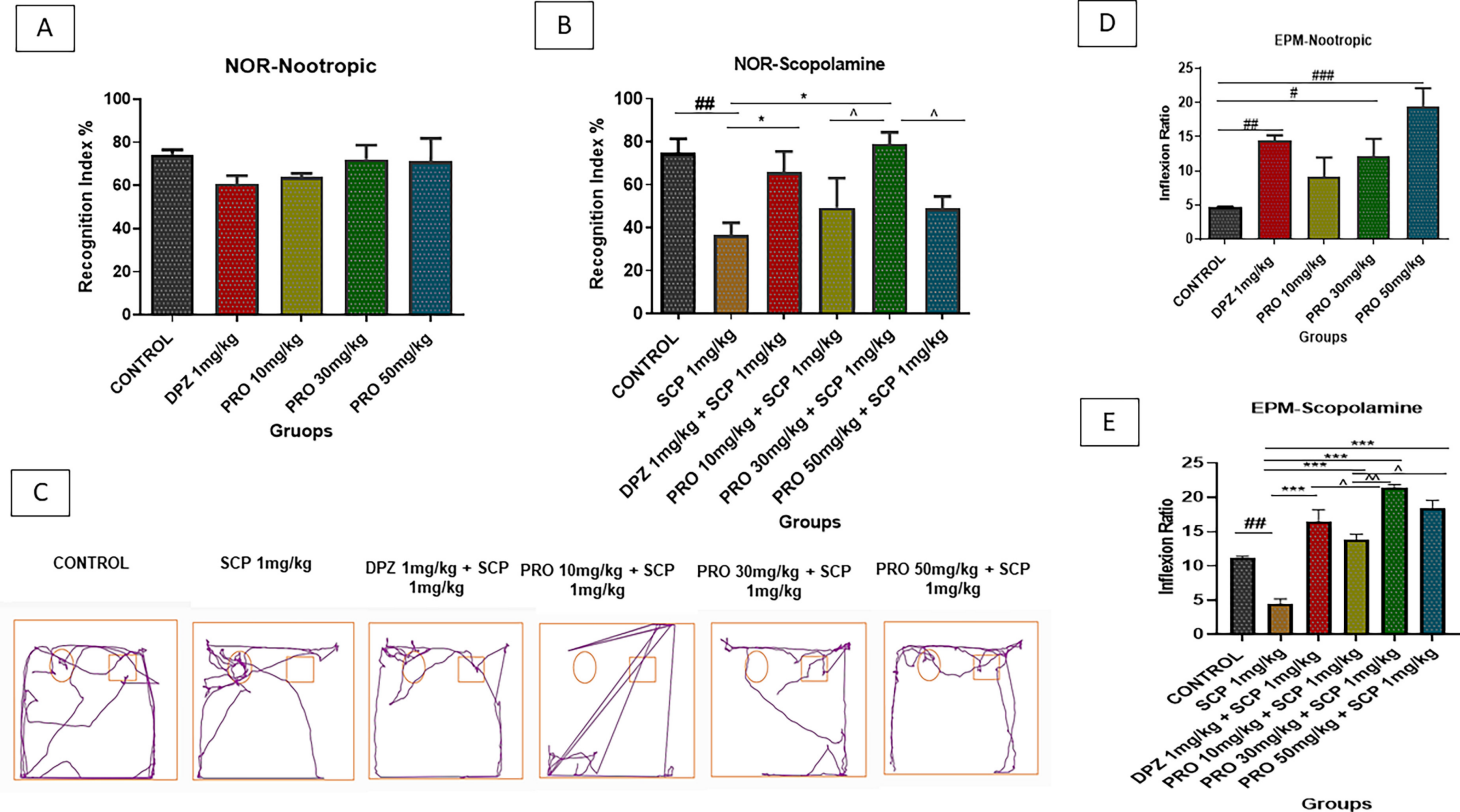
The findings demonstrate memory-related changes shown by PRO against SCP in behavioral analyses conducted by NOR and EPM. **(A)** Represents the graph plot for the recognition indices percentage in NOR for the nootropic model. The data suggest that the pretreated group of PRO showed an increase in the recognition index for NOR compared with the positive control DPZ (1 mg/kg b.wt.). However, no statistically significant difference was found in the nootropic model of DPZ (1 mg/kg b.wt.) and PRO (10 mg/kg, 30 mg/kg, and 50 mg/kg b.wt.) in between or as compared to the control group. **(B)** PRO enhances the long-term recognition indices percentage in the SCP-induced rat model. The bar graph represents the increase in the recognition indices percentage in NOR for the SCP (1 mg/kg. b.wt.) model, where statistically significant differences in recognition percentage of novel object exploration were seen in the control vs. SCP (1 mg/kg. b.wt.) model, ##*p* < 0.01, and a statistically significant difference is more between the positive control DPZ (1 mg/kg. b.wt.) and PRO (30 mg/kg. b.wt.) vs. SCP (1 mg/kg b.wt.), *p < 0.05. Moreover, the medium dose of PRO (30 mg/kg b.wt.) is statistically more significant as compared to PRO (10 mg/kg and 50 mg/kg b.wt.), ^∧^*p* < 0.05. **(C)** Represents exploratory track plot of the control, SCP (1 mg/kg b.wt.), DPZ (1 mg/kg b.wt.), and PRO (10 mg/kg, 30 mg/kg, and 50 mg/kg b.wt.) groups during a 5-min NOR test session generated from ANY-maze software. The statistical analysis by ANOVA (one-way) repeated measures with uncorrected Fisher’s LSD multiple comparison test and data are expressed as mean ± SEM, *n* = 3. **(D)** PRO restores PFC from anxiety confirmed by elevated plus maze (EPM), where the graph plot represents the inflection ratio in EPM for the nootropic model. In EPM, the inflection ratio was significantly increased at a medium and higher dose of PRO (30 mg/kg and 50 mg/kg b.wt.) and also for positive control DPZ (1 mg/kg) when compared to the control group, #*p* < 0.05, ###*p* < 0.001 and ##*p* < 0.01. **(E)** Represents the graph plot for the inflection ratio in EPM for the SCP model. In EPM, inflection ratio analysis showed that there was a significant difference between the control and SCP (1 mg/kg b.wt.), ##*p* < 0.01. Moreover, a significant difference was also found between DPZ (1 mg/kg b.wt.), PRO (10 mg/kg, 30 mg/kg, and 50 mg/kg b.wt.) vs. SCP (1 mg/kg b.wt.), ***p < 0.001. Also, a significant difference was found between positive control DPZ (1 mg/kg b.wt.) vs. PRO (10 mg/kg b.wt.), ^∧^*p* < 0.05. Medium and higher doses of PRO (30 mg/kg and 50 mg/kg b.wt.) showed significant increases against lower doses of PRO (10 mg/kg b.wt.), ^∧∧^*p* < 0.01 and ^∧^*p* < 0.05. Data suggest that anti-anxiety behavior is enhanced in medium and higher doses of PRO (30 mg/kg and 50 mg/kg b.wt.), as it showed a higher and comparable inflection ratio when compared to positive control DPZ (1 mg/kg b.wt.) and a significant increase against SCP (1 mg/kg b.wt.). Mean ± SEM, *n* = 3, and statistical analysis by one-way ANOVA followed by uncorrected Fisher’s LSD multiple comparison test.

Novel Object Recognition was applied as a behavior model to evaluate learning and memory, and evaluation was done during a 5-min NOR test session generated from ANY-MAZE software. The effect of different PRO (10 mg/kg, 30 mg/kg, and 50 mg/kg b.wt.) doses on memory function was assessed following 7 days of pre-treatment. The result was expressed as a recognition index (%) for the novel object. Based on the outcomes, the pretreatment groups of PRO (10 mg/kg, 30 mg/kg, and 50 mg/kg b.wt.) showed no significant changes as compared to the control and positive control DPZ groups (1 mg/kg b.wt.) (Figure 2A). The NOR test showed a reduction in recognition index (%) in the chronic SCP (1 mg/kg; b.wt.) model (Figure 2B). DPZ (1 mg/kg; b.wt.) and PRO (30 mg/kg b.wt.) are found to be more significant than negative control SCP (1 mg/kg b.wt.), *p < 0.05. Moreover, the medium dose of PRO (30 mg/kg b.wt.) is statistically more significant as compared to the lower and higher doses of PRO (10 mg/kg and 50 mg/kg b.wt.), ^∧^p < 0.05. The exploratory behavior track plot of animals also supports the neurobehavioral analysis (Figure 2C). It is thus inferred that PRO (30 mg/kg b.wt.) induces memory restoration in the SCP-induced model. The statistical analysis was done by ANOVA (one-way) repeated measures with an uncorrected Fisher’s LSD multiple comparison test, and the data are expressed as mean ± SEM, *n* = 3. EPM was applied to evaluate anti-anxiety behavior in rats. PRO restores PFC from anxiety confirmed by EPM, where the graph plot represents the inflection ratio in EPM for the nootropic model (Figure 2D). In EPM, the inflection ratio was significantly increased at a medium and higher dose of PRO (30 mg/kg and 50 mg/kg b.wt.) and also for positive control DPZ (1 mg/kg) when compared to the control group, #*p* < 0.05, ###*p* < 0.001 and ##*p* < 0.01. Figure 2E represents the graph plot for the inflection ratio in EPM for the SCP model. In EPM, inflection ratio analysis showed that there was a significant difference between the control and SCP (1 mg/kg b.wt.), ##*p* < 0.01. Moreover, a significant difference was also found between DPZ (1 mg/kg; b.wt.), PRO (10 mg/kg, 30 mg/kg, and 50 mg/kg b.wt.) vs. SCP (1 mg/kg b.wt.), ***p < 0.001. Also, a significant difference was found between positive control DPZ (1 mg/kg b.wt.) vs. PRO (10 mg/kg b.wt.), ^∧^*p* < 0.05. Medium and higher doses of PRO (30 mg/kg and 50 mg/kg b.wt.) showed significant increases against lower doses of PRO (10 mg/kg b.wt.), ^∧∧^*p* < 0.01 and ^∧^*p* < 0.05. Data suggest that anti-anxiety behavior is enhanced in medium and higher doses of PRO (30 mg/kg and 50 mg/kg b.wt.), as it showed a higher and comparable inflection ratio when compared to positive control DPZ (1 mg/kg b.wt.) and a significant increase against SCP (1 mg/kg b.wt.). Mean ± SEM, *n* = 3, and statistical analysis by one-way ANOVA followed by an uncorrected Fischer’s LSD multiple comparison test. PRO rescues from amnesia and restores memory markers through various signaling cascades.

### 3.2 PRO induces synthesis of memory markers

#### 3.2.1 PRO activates CaMKII and further increases CREB expression

**FIGURE 3:**
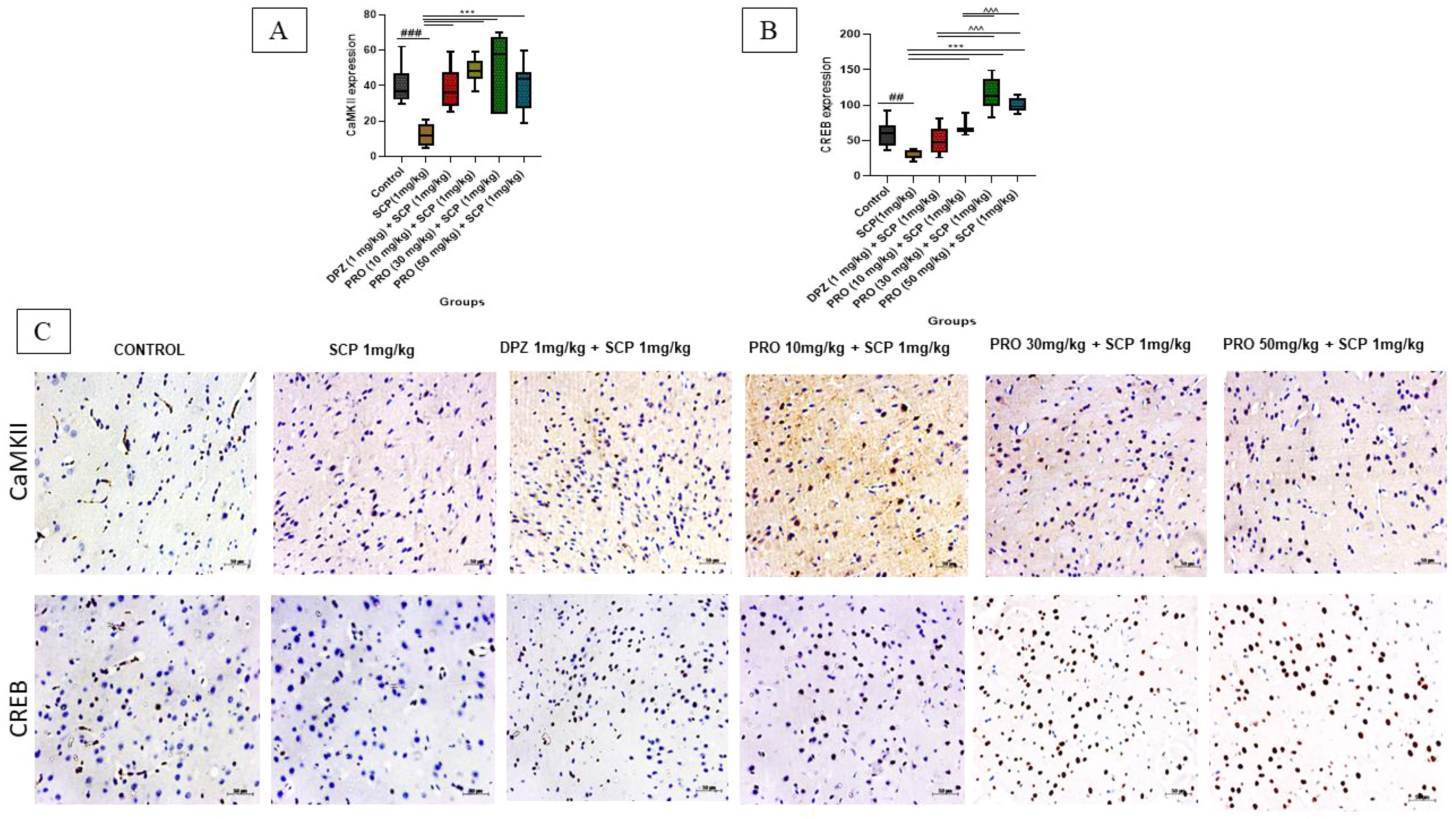
Propranolol (PRO) administration induces the CaMKII shuttle Ca^2+^/CaM to initiate CREB phosphorylation, which in turn aids in the long-term neuronal plasticity in the PFC against an SCP-induced rat model. CaMKII activation mediates the exocytosis on the postsynaptic surface, thus further enhancing synaptic strength and increasing the activation of synapses for neuronal signaling. The brain’s long-term memory formation and neural plasticity are both known to be influenced by CREB. **(A)** Represents the quantification of CaMKII, where SCP (1 mg/kg b.wt.) shows a significantly lower expression of CaMKII as compared to the control, ###*p* < 0.001. Also, DPZ (1 mg/kg b.wt.) and PRO (10 mg/kg, 30 mg/kg, and 50 mg/kg b.wt.) show significantly higher expression of CaMKII than SCP (1 mg/kg b.wt.), ***p < 0.001. Min to Max, *n* = 9, and statistical analysis by one-way ANOVA followed by Tukey’s multiple comparison test. **(B)** Quantification of CREB expression shows statistically significant higher expression of control when compared to SCP (1 mg/kg b.wt.), ##*p* < 0.01. All doses of PRO (10 mg/kg, 30 mg/kg, and 50 mg/kg b.wt.) and positive control DPZ (1 mg/kg b.wt.) show significantly higher expression when compared to SCP (1 mg/kg b.wt.), ***p < 0.001. PRO (30 mg/kg, and 50 mg/kg b.wt.) had a significantly higher expression when compared to DPZ (1 mg/kg b.wt.), ^∧∧∧^*p* < 0.001. PRO (30 mg/kg and 50 mg/kg, b.wt.) were more significant than PRO (10 mg/kg, b.wt.), ^∧∧∧^*p* < 0.001. Min to Max, *n* = 9, and statistical analysis by one-way ANOVA followed by Tukey’s multiple comparison test. **(C)** Represents the photomicrographs of CaMKII/ CREB expression of SCP (1 mg/kg b.wt.) shows lower expression as compared to control, positive control DPZ (1 mg/kg b.wt.), and PRO (10 mg/kg, 30 mg/kg, and 50 mg/kg b.wt.). Scale bar = 50 μm (CaMKII/CREB immunostaining 20×).

The effect of chronic administration of PRO against SCP-induced memory and possible PFC CaMKII deregulation was investigated in rats. PRO enhances synaptic strength by activating the CaMKII receptor in an SCP-induced rat model, which directly and indirectly affects other memory markers like CREB-BDNF and PKMζ. CaMKII expression shows that SCP (1 mg/kg b.wt.) shows a significantly lower expression of CaMKII as compared to the control, ###*p* < 0.001. Also, DPZ (1 mg/kg; b.wt.), PRO (10 mg/kg, 30 mg/kg, and 50 mg/kg b.wt.) show significantly higher expression of CaMKII against SCP (1 mg/kg b.wt.), ***p < 0.001 (Figure 3A). Min to Max, *n* = 9, and statistical analysis by one-way ANOVA followed by Tukey’s multiple comparison test. Moreover, CaMKII activation mediates the exocytosis on the postsynaptic surface, thus further enhancing synaptic strength and increasing the activation of synapses for neuronal signaling (Kennedy and Ehlers, 2011). Further CaMKII shuttle Ca^2+^/CaM to initiate CREB phosphorylation, which in turn aids in the long-term neuronal plasticity in the PFC against a SCP-induced rat model. PRO also activates CREB expression in the SCP-induced model. CREB-lower expression was observed for the SCP (1 mg/kg b.wt.) group. CREB expression shows statistically significantly higher expression of control when compared to SCP (1 mg/kg b.wt.), ##*p* < 0.01. All doses of PRO (10 mg/kg, 30 mg/kg, and 50 mg/kg b.wt.) and positive control DPZ (1 mg/kg b.wt.) show significantly higher expression when compared to SCP (1 mg/kg b.wt.), ***p < 0.001. PRO (30 mg/kg and 50 mg/kg b.wt.) had a significantly higher expression when compared to DPZ (1 mg/kg b.wt.), ^∧∧∧^*p* < 0.001. PRO (30 mg/kg and 50 mg/kg, b.wt.) were more significant than PRO (10 mg/kg, b.wt.), ^∧∧∧^*p* < 0.001 (Figure 3B). Min to Max, *n* = 9, and statistical analysis by one-way ANOVA followed by Tukey’s multiple comparison test. IHC images of CaMKII/CREB expression of SCP (1 mg/kg; b.wt.) show lower expression as compared to control, positive control DPZ (1 mg/kg; b.wt.), PRO (10 mg/kg, 30 mg/kg, and 50 mg/kg b.wt.). Scale bar = 50 μm (CaMKII/CREB immunostaining 20×). Activation of CREB in PRO groups as multiple exposures of SCP down-regulate the expression, which is also confirmed by CaMKII expression (Figure 3C).

#### 3.2.2 PRO restores BDNF synthesis

**FIGURE 4:**
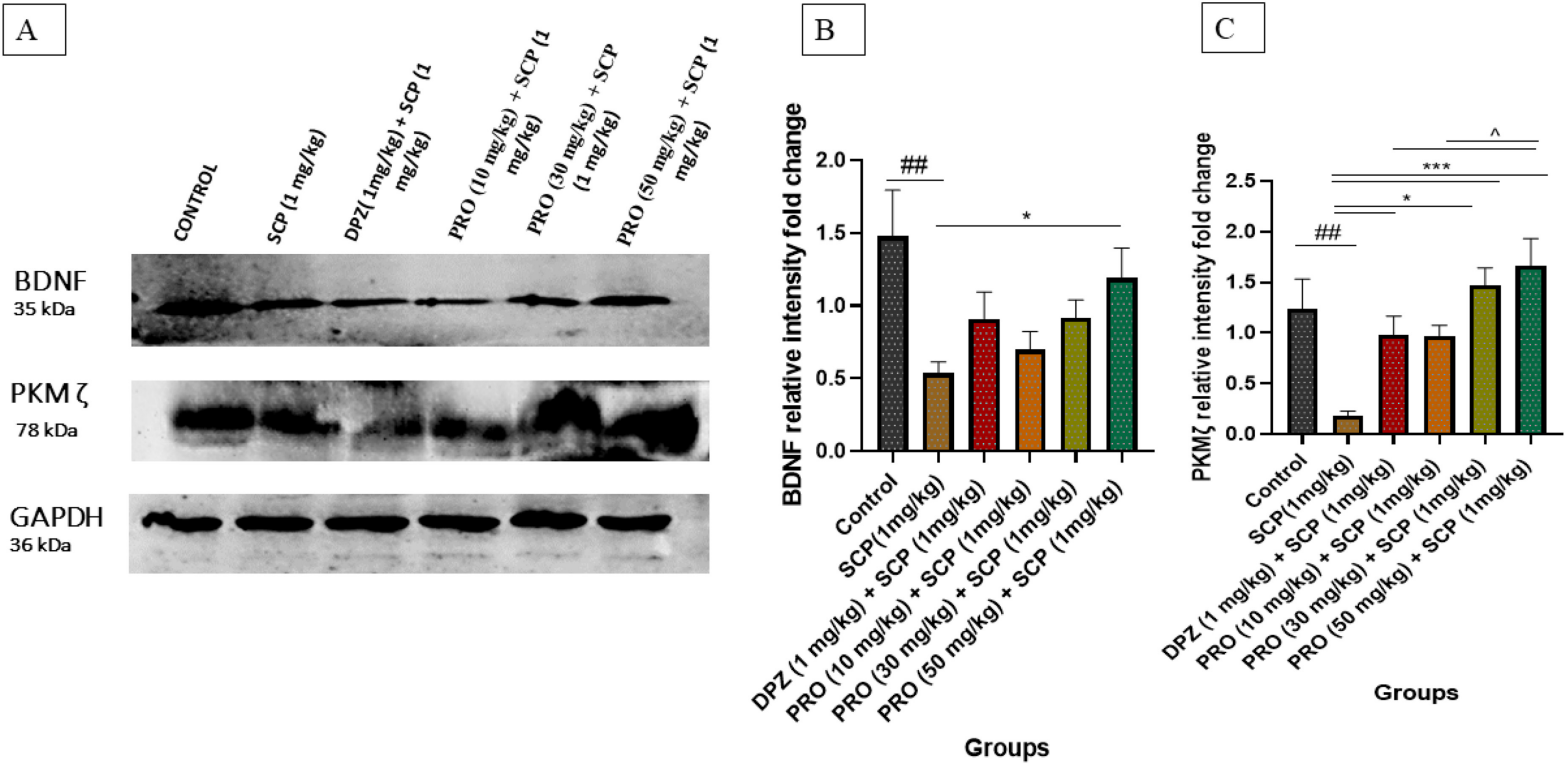
In a SCP-induced rat model, PRO increases the synthesis of molecular markers related to plasticity, including BDNF and PKMζ, in the PFC region. BDNF is a promising target for the treatment of cognitive deficits and anxiety-related memory disorders. Similarly, research on the enduring stability of memory has revealed that protein kinase Mzeta (PKMζ) plays a potentially crucial function in preserving established memory. BDNF, in turn, increases synaptic effectiveness via protein kinase Mζ (PKMζ), potentially protecting long-term memory. **(A)** Represent the BDNF and PKMζ expression through western blot in all the experimental groups. GAPDH was used as the normalizing factor for BDNF and PKMζ relative intensity fold change. **(B)** Represent an expression of BDNF, where significantly lower expression of BDNF was observed for the SCP (1 mg/kg b.wt.) group vs. control, ##*p* < 0.01. PRO (50 mg/kg b.wt.) shows significantly higher expression of BDNF when compared to SCP (1 mg/kg b.wt.), *p < 0.05. No significant differences were found in the expression of BDNF after administration of positive control DPZ (1 mg/kg b.wt.) and PRO (10 mg/kg and 30 mg/kg b.wt.). **(C)** Represent an expression of PKMζ, where significantly lower expression of PKMζ was observed for the SCP (1 mg/kg b.wt.) group when compared to control, ##*p* < 0.01. Positive control DPZ (1 mg/kg b.wt.) and PRO (10 mg/kg b.wt.) show significant enhancement in the PKMζ expression when compared to SCP (1 mg/kg b.wt.), *p < 0.05. Also, medium and higher doses of PRO (30 mg/kg and 50 mg/kg, b.wt.) show a significant threefold higher expression of PKMζ when compared to SCP (1 mg/kg, b.wt.), ***p < 0.001. Moreover, PRO (50 mg/kg, b.wt.) is significant and found to be higher in PKMζ expression when compared to DPZ (1 mg/kg, b.wt.) and PRO (10 mg/kg, b.wt.), ^∧^*p* < 0.05. Overall data **(A–C)** suggest strong induction of BDNF and PKMζ synthesis in the PFC of the rat brain after administration of PRO (50 mg/kg, b.wt.) for BDNF and PRO (30 mg/kg and 50 mg/kg, b.wt.) for PKMζ. Mean ± SEM, *n* = 3, and statistical analysis by one-way ANOVA followed by uncorrected Fisher’s LSD multiple comparison test.

Brain derived neurotrophic factor is a molecular marker widely studied for its role in the memory and learning. A significantly lower expression of BDNF was observed for the SCP (1 mg/kg b.wt.) group vs. control, ##*p* < 0.01. PRO (50 mg/kg b.wt.) shows significantly higher expression of BDNF when compared to SCP (1 mg/kg b.wt.), *p < 0.05. No significant differences were found in the expression of BDNF after administration of positive control DPZ (1 mg/kg; b.wt.) and PRO (10 mg/kg and 30 mg/kg; b.wt.) (Figure 4B). Research has also established the role of BDNF in long-term memory restoration. Furthermore, studies established the functionalities of BDNF after PRO exposure in the PFC region (Rosas-Vidal et al., 2014). GAPDH was used as the normalizing factor for BDNF and PKMζ, relative intensity fold change (Figure 4A).

#### 3.2.3 PRO confirms the activation of PKMζ

Protein kinase Mζ showed a significantly lower expression in the SCP (1 mg/kg b.wt.) group when compared to the control, ##*p* < 0.01. Positive control DPZ (1 mg/kg b.wt.) and PRO (10 mg/kg b.wt.) show significant enhancement in the PKMζ expression when compared to SCP (1 mg/kg b.wt.), *p < 0.05. Also, medium and higher doses of PRO (30 mg/kg and 50 mg/kg, b.wt.) show a significant threefold higher expression of PKMζ when compared to SCP (1 mg/kg, b.wt.), ***p < 0.001. Moreover, PRO (50 mg/kg, b.wt.) is significant and found to be higher in PKMζ expression when compared to DPZ (1 mg/kg, b.wt.) and PRO (10 mg/kg, b.wt.), ^∧^*p* < 0.05 (Figure 4C). One-way ANOVA statistical analysis followed by an uncorrected Fischer’s LSD multiple comparison test was used, Mean ± SEM, *n* = 3. GAPDH was used as the normalizing factor for BDNF and PKMζ, relative intensity fold change (Figure 4A and Supplementary Data Sheet 1).

Overall data suggest strong induction of memory formation after BDNF and PKMζ synthesis in the PFC of the rat brain after administration of PRO (50 mg/kg, b.wt.) for BDNF and PRO (30 mg/kg and 50 mg/kg, b.wt.) for PKMζ.

#### 3.2.4 PRO administration reduces mitochondria ROS production and restores the MMP (▲ψm)

**FIGURE 5:**
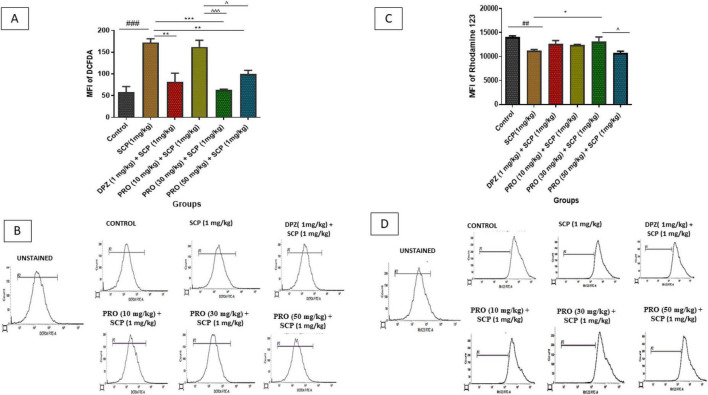
Propranolol (PRO) administration reduces mitochondrial ROS production and restores the MMP (▲ψm) measured through flow cytometry in a SCP-induced model **(A)** in the left panel upper portion represents the bar graph depicting the mean fluorescence intensity (MFI) of mitochondrial ROS production, where a mitochondrial ROS was found statistically more significant in SCP (1 mg/kg b.wt.) vs. control, ###*P* < 0.001. Mitochondrial ROS were significantly reduced in PRO (30 mg/kg) when compared to SCP (1 mg/kg b.wt.), ***p < 0.001. Also, positive control DPZ (1 mg/kg b.wt.) and PRO (50 mg/kg b.wt.) were found to significantly lower mitochondrial ROS production when compared to SCP (1 mg/kg b.wt.), **p < 0.01. Moreover, PRO (30 mg/kg and 50 mg/kg b.wt.) also showed significant changes as compared to PRO (10 mg/kg b.wt.), ^∧^*p* < 0.05, ^∧∧∧^*p* < 0.001. Mean ± SEM, *n* = 4, and statistical analysis by one-way ANOVA followed by Tukey’s multiple comparison test. **(B)** Left panel lower portion of DCFDA shows the representative images of histograms of the unstained, control, SCP (1 mg/kg b.wt.) group, positive control DPZ (1 mg/kg b.wt.), and PRO (10 mg/kg, 30 mg/kg, and 50 mg/kg b.wt.) groups. **(C)** PRO modulates the ▲ψm in a SCP-induced model of rats. ▲ψm is statistically reduced significantly in the SCP (1 mg/kg, b.wt.) vs. the control group, ##*p* < 0.01. PRO (30 mg/kg, b.wt.) shows a significant increase in mitochondrial membrane potential when compared to SCP (1 mg/kg, b.wt.) and also against PRO (50 mg/kg, b.wt.), *p < 0.05, ^∧^*p* < 0.05. Data indicate that PRO (30 mg/kg, b.wt.) shows increased mitochondrial membrane potential and has a protective effect against SCP (1 mg/kg, b.wt.) mediated toxicity when compared to the positive control DPZ (1 mg/kg, b.wt.) and PRO (10 mg/kg and 50 mg/kg, b.wt.). Mean ± SEM, *n* = 2, and statistical analysis by one-way ANOVA followed by uncorrected Fisher’s LSD multiple comparison test. **(D)** Represents ▲ψm showing the histograms of unstained, control, SCP (1 mg/kg b.wt.), positive control DPZ (1 mg/kg b.wt.), and PRO (10 mg/kg, 30 mg/kg, and 50 mg/kg b.wt.) through Rhodamine 123 dye. ^∧^*p* < 0.05, ^∧∧^*p* < 0.01, ^∧∧∧^*p* < 0.001 indicate comparison with the PRO (10 mg/kg) group.

After confirming the memory restoration by PRO, mitochondrial dynamics were also taken into consideration. PRO administration lowers mitochondrial ROS production in a SCP-induced model. The mean fluorescence intensity (MFI) of mitochondrial ROS production was significantly higher in SCP (1 mg/kg b.wt.) vs. control ### *p* < 0.001. mitochondrial ROS were significantly reduced in PRO (30 mg/kg) when compared to SCP (1 mg/kg b.wt.), ***p < 0.001. Also, positive control DPZ (1 mg/kg b.wt.) and PRO (50 mg/kg b.wt.) were found to significantly lower mitochondrial ROS production when compared to SCP (1 mg/kg b.wt.), **p < 0.01. Moreover, PRO (30 mg/kg and 50 mg/kg b.wt.) also showed significant changes as compared to PRO (10 mg/kg b.wt.), ^∧∧^*p* < 0.01, ^∧^*p* < 0.05 (Figure 5A). One-way ANOVA followed by Turkey’s multiple comparison test was used, Mean ± SEM, *n* = 4. The DCFDA histograms of the control, positive control DPZ (1 mg/kg b.wt.), and PRO (10 mg/kg, 30 mg/kg, and 50 mg/kg b.wt.) groups show a decrease in mitochondrial ROS production as compared to the SCP (1 mg/kg b.wt.) group (Figure 5B). PRO modulates the ▲ψm in a SCP-induced model of rats. ▲ψm is statistically reduced significantly in the SCP (1 mg/kg, b.wt.) vs. the control group, ##*p* < 0.01. PRO (30 mg/kg, b.wt.) shows a significant increase in mitochondrial membrane potential when compared to SCP (1 mg/kg, b.wt.) and also against PRO (50 mg/kg, b.wt.), *p < 0.05, ^∧^*p* < 0.05. Data indicate that PRO (30 mg/kg, b.wt.) shows increased mitochondrial membrane potential and has a protective effect against SCP (1 mg/kg, b.wt.)-mediated toxicity when compared to the positive control DPZ (1 mg/kg, b.wt.) and PRO (10 mg/kg and 50 mg/kg, b.wt.) (Figure 5C). Mean ± SEM, *n* = 2, and statistical analysis by one-way ANOVA followed by an uncorrected Fischer’ LSD multiple comparison test. (D) Represents ▲ψm showing the histograms of unstained, control, SCP (1 mg/kg b.wt.), positive control DPZ (1 mg/kg b.wt.), and PRO (10 mg/kg, 30 mg/kg and 50 mg/kg b.wt.) through Rhodamine 123 dye (Figure 5D).

### 3.3 PRO reduces SCP-mediated Aβ_1–42_ plaque deposition and inhibits the inflammatory markers TNFα and GFAP

**FIGURE 6:**
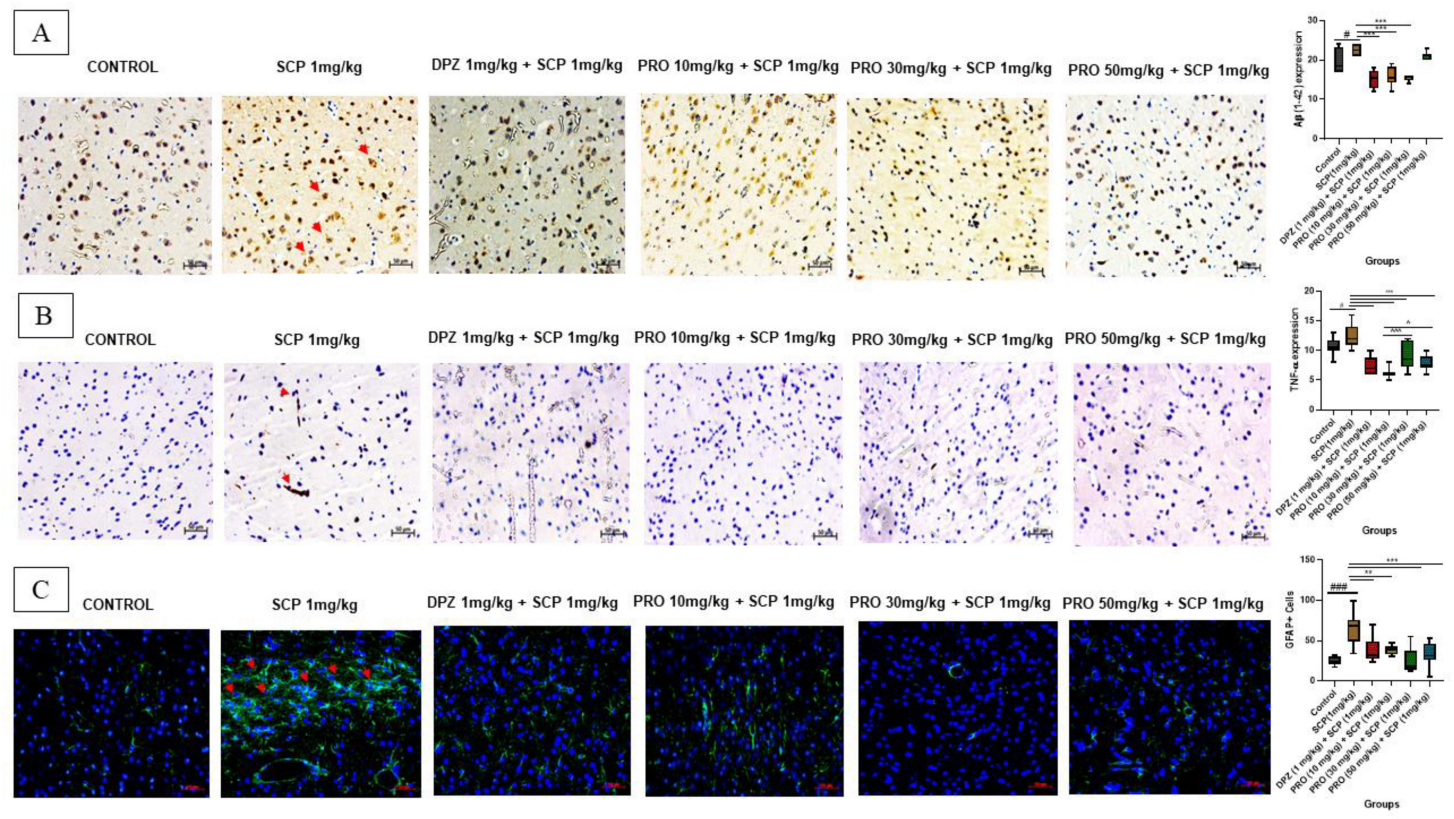
Propranolol (PRO) administration mitigated the toxicity and inflammation associated with SCP mediated rat model. **(A)** Represents IHC images and showing inhibitory effect of PRO against SCP-indued β-amyloid 1-42 (Aβ1-42) plaque deposition in the PFC. Aβ1-42 plaque deposition is higher in the SCP (1 mg/kg b.wt.) group, which exhibited intraneuronal Aβ acclamation (red arrow) and later progressed to Aβ plague deposition. Scale Bar = 50 μm (Aβ immunostaining 20×) and graph depicting the quantification of Aβ plaque, where statistically significant differences were observed between control and SCP (1 mg/kg b.wt.), #*p* < 0.05. Also, significant decreases in Aβ deposition were seen in positive control DPZ (1 mg/kg b.wt.) and PRO (10 mg/kg and 30 mg/kg b.wt.) when compared to SCP (1 mg/kg b.wt.), ***p < 0.001. Min to Max, *n* = 6, and statistical analysis by one-way ANOVA followed by uncorrected Fisher’s LSD multiple comparison test. **(B)** PRO lowers the expression of the proinflammatory marker tumor necrosis factor-alpha (TNF-α) in the PFC of the SCP-induces rat model. Photomicrographs of pro-inflammatory cytokine TNF-α showing the higher expression in SCP (1 mg/kg b.wt.) when compared to control and all the treatment groups of positive control DPZ (1 mg/kg b.wt.) and PRO (10 mg/kg, 30 mg/kg, and 50 mg/kg b.wt.). Scale Bar = 50 μm (TNF-α immunostaining 20×). Graph showing the quantification of TNF-α expression, also considered to be an initiator of inflammation. A statistically significant higher expression of TNF-α was recorded for SCP (1 mg/kg b.wt.) vs. control, #*p* < 0.05. Also, significantly lower expression of TNF-α deposition was seen in positive control DPZ (1 mg/kg b.wt.) and PRO (10 mg/kg, 50 mg/kg, and 30 mg/kg b.wt.) vs. SCP (1 mg/kg b.wt.), ***p < 0.001. Lower and higher doses of PRO (10 mg/kg and 50 mg/kg b.wt.) showed significant decreases against medium doses of PRO (30 mg/kg b.wt.), ^∧∧∧^*p* < 0.001 and ^∧^*p* < 0.05. Min to Max, *n* = 8, and statistical analysis by one-way ANOVA followed by uncorrected Fisher’s LSD multiple comparison test. **(C)** PRO inhibits the activation of astrocytes in GFAP staining in the PFC in an SCP-induced model, where the photomicrographs of GFAP, where higher expression of GFAP-positive cells was seen in the SCP (1 mg/kg b.wt.) group when compared to control and all the treatment groups of positive control DPZ (1 mg/kg b.wt.) and PRO (10 mg/kg, 30 mg/kg, and 50 mg/kg b.wt.). Control, positive control DPZ (1 mg/kg, b.wt.), and PRO (10 mg/kg, 30 mg/kg, and 50 mg/kg, b.wt.) groups exhibited small-sized astrocytes in the granule and neuropil of the PFC region of the rat brain as compared to SCP (1 mg/kg, b.wt.), where large astrocytes with thick multiple-ramified processes (arrowhead) were seen. Scale bar = 50 μm (GFAP immunostaining 20×). Graph showing the quantification of GFAP-positive cells, where statistically significant GFAP-positive cells were seen in SCP (1 mg/kg b.wt.) vs. control ###*p* < 0.001. Moreover, significantly lower expression of GFAP-positive cells was also seen in positive control DPZ (1 mg/kg b.wt.) and PRO (10 mg/kg, 30 mg/kg, and 50 mg/kg b.wt.), **p < 0.01, ***p < 0.001 vs. scopolamine (1 mg/kg b.wt.). Min to Max, *n* = 9, and statistical analysis by one-way ANOVA followed by Tukey’s multiple comparison test.

Propranolol administration mitigated Aβ_1–42_-mediated toxicity and inflammation associated with the SCP-mediated rat model. IHC images show the inhibitory effect of PRO against SCP-indued β-amyloid 1-42 (Aβ1-42) plaque deposition in the PFC. Aβ1-42 plaque deposition is higher in the SCP (1 mg/kg b.wt.) group, which exhibited intraneuronal Aβ accumulation (red arrow) and later progressed to Aβ plague deposition. Scale Bar = 50 μm (Aβ immunostaining 20×) and graph depicting the quantification of Aβ plaque, where statistically significant differences were observed between control and SCP (1 mg/kg b.wt.), #*p* < 0.05. Also, significant decreases in Aβ deposition were seen in positive control DPZ (1 mg/kg b.wt.) and PRO (10 mg/kg and 30 mg/kg b.wt.) when compared to SCP (1 mg/kg b.wt.), ***p < 0.001 (Figure 6A). Min to Max, *n* = 6, and statistical analysis by one-way ANOVA followed by an uncorrected Fischer’s LSD multiple comparison test. After confirming the mitigation of Aβ_1–42_ plaque by PRO, the proinflammatory marker TNF-α was also evaluated in the PFC of the SCP-induces rat model. Photomicrographs of pro-inflammatory cytokine TNF-α show higher expression in SCP (1 mg/kg b.wt.) when compared to control and all the treatment groups of positive control DPZ (1 mg/kg b.wt.) and PRO (10 mg/kg, 30 mg/kg, and 50 mg/kg b.wt.). Scale bar = 50 μm (TNF-α immunostaining 20×). A graph showing the quantification of TNF-α expression is also considered to be an initiator of inflammation. A statistically significant higher expression of TNF-α was recorded for SCP (1 mg/kg b.wt.) vs. control, #*p* < 0.05. Also, significantly lower expression of TNF-α deposition was seen in positive control DPZ (1 mg/kg b.wt.) and PRO (10 mg/kg, 50 mg/kg, and 30 mg/kg b.wt.) vs. SCP (1 mg/kg b.wt.), ***p < 0.001. Lower and higher doses of PRO (10 mg/kg and 50 mg/kg b.wt.) showed significant decreases against medium doses of PRO (30 mg/kg b.wt.), ^∧∧∧^*p* < 0.001 and ^∧^*p* < 0.05 (Figure 6B). Min to Max, *n* = 8, and statistical analysis by one-way ANOVA followed by an uncorrected Fisher’s LSD multiple comparison test. Moreover, after confirming the inhibitory effect of PRO against Aβ_1–42_ and TNF-α, PRO inhibits the activation of astrocytes in GFAP staining in the PFC in a SCP-induced model, where the photomicrographs of GFAP show higher expression of GFAP-positive cells in the SCP (1 mg/kg b.wt.) group when compared to control and all the treatment groups of positive control DPZ (1 mg/kg b.wt.) and PRO (10 mg/kg, 30 mg/kg and 50 mg/kg b.wt.). Control, positive control DPZ (1 mg/kg b.wt.), and PRO (10 mg/kg, 30 mg/kg, and 50 mg/kg b.wt.), groups exhibited small-sized astrocytes in the granule and neuropil of the PFC region of the rat brain as compared to SCP (1 mg/kg, b.wt.), where large astrocytes with thick multiple-ramified processes (arrowhead) were seen. Scale bar = 50 μm (GFAP immunostaining 20×). Graph showing the quantification of GFAP-positive cells, where statistically significant GFAP-positive cells were seen in SCP (1 mg/kg b.wt.) vs. control ###*p* < 0.001. Moreover, significantly lower expression of GFAP-positive cells was also seen in positive control DPZ (1 mg/kg b.wt.) and PRO (10 mg/kg, 30 mg/kg, and 50 mg/kg b.wt.), **p < 0.01, ***p < 0.001 vs. SCP (1 mg/kg b.wt.) (Figure 6C). Min to Max, *n* = 9, and statistical analysis by one-way ANOVA followed by Tukey’s multiple comparison test.

### 3.4 PRO treatment attenuates neuronal damage by restoring tissue pathophysiology

**FIGURE 7:**
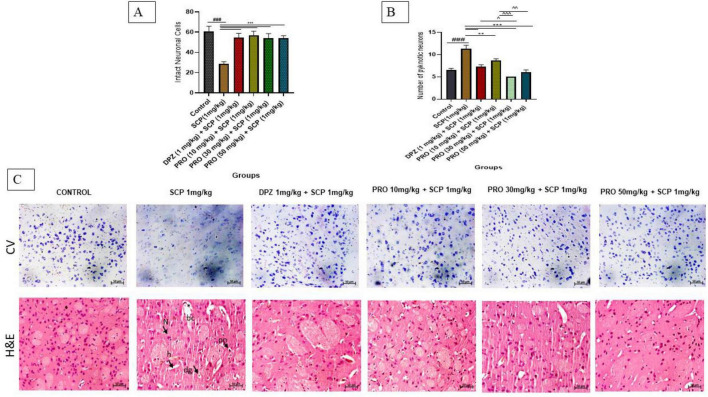
Propranolol (PRO) treatment restores the pathophysiological structure of PFC in the SCP-induced rat model. **(A)** Shows the quantification of intact neurons by CV stain, where statistically significant differences are seen between control and SCP (1 mg/kg b.wt.), ###*p* < 0.001. Also, the number of intact neurons is significantly higher in positive control DPZ (1 mg/kg b.wt.) and all doses of PRO (10 mg/kg, 30 mg/kg, and 50 mg/kg b.wt.) as compared to SCP (1 mg/kg b.wt.), ***p < 0.001. Mean ± SEM, *n* = 10, and statistical analysis by one-way ANOVA followed by Tukey’s multiple comparison test. **(B)** Represents the quantification of pyknotic neurons through H&E staining, where SCP (1 mg/kg b.wt.) shows a significant increase in the number of pyknotic neurons as compared to the control, ###*p* < 0.001. Also, a significant reduction of pyknotic neurons was seen in positive control DPZ (1 mg/kg b.wt.) and PRO (10 mg/kg, 30 mg/kg, and 50 mg/kg b.wt.) vs. SCP (1 mg/kg b.wt.), **p < 0.01, ***p < 0.001. PRO (30 mg/kg, b.wt.) showed a more significant reduction of pyknotic nuclei when compared to the positive control DPZ (1 mg/kg, b.wt.), ^∧^*p* < 0.05. Moreover, PRO (30 mg/kg and 50 mg/kg, b.wt.) showed a significantly reduced number of pyknotic neurons when compared to PRO (10 mg/kg, b.wt.), ^∧∧^*p* < 0.01, ^∧∧∧^*p* < 0.001. Mean ± SEM, *n* = 6, and statistical analysis by one-way ANOVA followed by Tukey’s multiple comparison test. **(C)** Represents the photomicrographs of cresyl violet (CV)/hematoxylin and eosin (H&E) staining. Intact and organized neuronal structures are visible for the control and all treatment groups through CV. Photomicrographs of H&E staining shows disruption in the structure of the SCP (1 mg/kg b.wt.) group where a large number of dilated blood capillaries (*bc) were observed. Also, pyknotic nuclei (pn), pericellular haloes (h), and deeply (dg) stained glial cell nuclei can be noticed in the vacuolated neutrophil. Normal pyramidal cells (N) are hardly seen in the SCP (1 mg/kg b.wt.) group when compared to the control, positive control DPZ (1 mg/kg b.wt.), and PRO (10 mg/kg, 30 mg/kg, and 50 mg/kg b.wt.). Scale bar = 50 μm (histology 20×). *p < 0.05, **p < 0.01, ***p < 0.001 indicate comparison with the SCP group; ^∧∧^*p* < 0.01 and ^∧∧∧^*p* < 0.001 indicate comparison with the PRO (10 mg/kg)

Propranolol treatment restores the neuronal structure in the PFC of a SCP-induced rat model. Intact neurons by CV stain, where statistically significant differences are seen between control and SCP (1 mg/kg b.wt.), ###*p* < 0.001. Also, the number of intact neurons is significantly higher in positive control DPZ (1 mg/kg b.wt.) and all doses of PRO (10 mg/kg, 30 mg/kg, and 50 mg/kg b.wt.) as compared to SCP (1 mg/kg b.wt.), ***p < 0.001 (Figure 7A). Mean ± SEM, *n* = 10, and statistical analysis by one-way ANOVA followed by Turkey’s multiple comparison test. Pyknotic neurons through H&E staining are confirmed in Figure 7B. SCP (1 mg/kg b.wt.) shows a significant increase in the number of pyknotic neurons as compared to the control, ###*p* < 0.001. Also, a significant reduction of pyknotic neurons was seen in positive control DPZ (1 mg/kg b.wt.) and PRO (10 mg/kg, 30 mg/kg, and 50 mg/kg b.wt.) vs. SCP (1 mg/kg b.wt.), ***p < 0.001. PRO (30 mg/kg, b.wt.) showed a more significant reduction of pyknotic nuclei when compared to the positive control DPZ (1 mg/kg, b.wt.), ^∧∧^*p* < 0.01. Moreover, PRO (30 mg/kg and 50 mg/kg, b.wt.) showed a significantly reduced number of pyknotic neurons when compared to PRO (10 mg/kg, b.wt.), ^∧∧∧^*p* < 0.001. Mean ± SEM, *n* = 6, and statistical analysis by one-way ANOVA followed by Turkey’s multiple comparison test. Photomicrographs of CV, and H&E staining also confirm the histological restoration by PRO (Figure 7C). The intact and organized neuronal structure is visible for the control and all treatment groups through CV. Photomicrographs of H&E staining show disruption in the structure of the SCP (1 mg/kg b.wt.) group, where a large number of dilated blood capillaries (*bc) were observed. Also, pyknotic nuclei (pn), pericellular haloes (h), and deeply (dg) stained glial cell nuclei can be noticed in the vacuolated neutrophils. Normal pyramidal cells (N) are hardly seen in the SCP (1 mg/kg b.wt.) group when compared to the control, positive control DPZ (1 mg/kg b.wt.) and PRO (10 mg/kg, 30 mg/kg, and 50 mg/kg b.wt.). Scale bar = 50 μm (histology 20×).

## 4 Discussion

This is the first study to investigate the effectiveness of different PRO concentrations (10 mg/kg, 30 mg/kg, and 50 mg/kg) versus DPZ, an FDA-approved drug used in AD clinical settings, as well as to examine the relative contributions of nootropic and anti-amnesic effects against SCP-mediated toxicity in PFC region of wistar rats. It has been determined that PRO triggers many intracellular neuronal signaling cascades that aid in the maintenance and reverse cognitive deficits associated with stress and anxiety (Aisa et al., 2007). Moreover, the exact role and mechanism of the development of long-term memory of PRO, however, are still uncertain. The cascades that strengthen Arc, activate CaMKIIα and trigger the CREB-BDNF and PKMζ dependent pathways are being investigated in this work. Importantly, the present investigation is designed to delineate associative molecular and behavioral changes accompanying propranolol treatment, rather than to establish definitive causal relationships between individual signaling components.

In the present study PRO exhibits an anti-amnesic effect through NOR; pretreatment with PRO reduced SCP-induced memory impairment, as seen by an increase in the recognition index percentage (Zeeshan et al., 2019). It has been found that recognition memory is significantly enhanced by a medium dosage of PRO. However, it was discovered that PRO exhibited nootropic effects in NOR behavior paradigms. In addition, stress, anxiety, depressive disorders, and a host of other symptoms are linked to the onset of AD. We also investigated the anti-anxiety behavior in wistar rats using EPM. When it came to the effects of nootropics in EPM, PRO demonstrated a much higher inflection ratio, whilst the SCP-induced group displayed similar results as seen in NOR. Thus, PRO seems to have an anti-anxiety (Steenen et al., 2016) and anti-amnesic effect. These behavioral outcomes support the functional relevance of propranolol under conditions of cholinergic disruption, while remaining consistent with an AD-like amnestic framework rather than a progressive neurodegenerative disease model.

**FIGURE 8:**
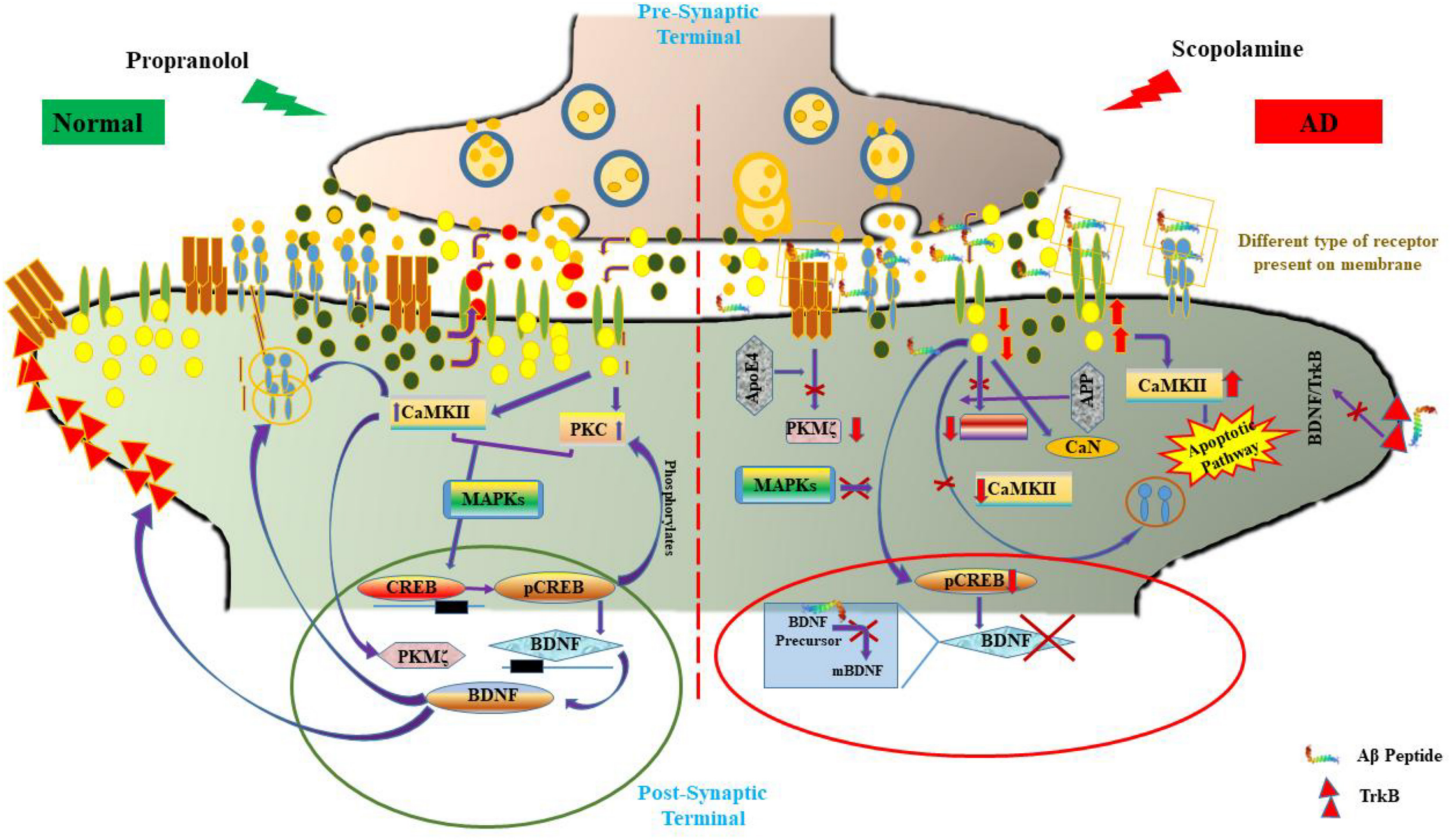
Diagrammatic depiction of the chemical cascade involved in AD and normal long-term memory consolidation through PRO. Normal Condition: By facilitating the exocytosis of several receptors on postsynaptic neurons’ surface, activation of CaMKII strengthens synapses by altering their plasticity. Furthermore, CaMKII promotes the phosphorylation of CREB via MAPKs and aids in the production of PKMζ. Further phosphorylation of PKC and increased BDNF transcription are caused by phosphorylated CREB, which also facilitates Arc protein translation. Furthermore, BDNF increases the trafficking of AMPA receptors to the cell surface and binds to its TrkB receptor, which is present at the surface. This increases synaptic strength and speeds up the consolidation of long-term memory. AD Condition: In contrast, binding of Aβ peptide to NMDA receptors causes these channels to open for a longer period and a greater Ca^2+^ influx, which overactivates CaMKII and triggers the apoptotic pathway. AD-related genes like APP also encourage Ca^2+^ to bind to calcineurin (CaN) rather than calmodulin (CaM), which facilitates LTD rather than LTP. By decreasing AMPA receptor trafficking to the cell surface, this route also weakens and lessens the plasticity and strength of synapses. In addition, CaN binding of Ca^2+^ decreases the expression of Arc in AD and downregulates the CREB-BDNF pathway. The insulin signaling-dependent PKMζ production is also impaired by Aβ peptides and the AD-related gene ApoE4, which downregulates its expression in AD and contributes to memory impairment.

To validate the behavior task results, we also looked at the unique memory signaling cascade. More recent research indicates that CaMKII plays a more significant role in long-term memory consolidation and storage (Yasuda et al., 2022). Here, we found that SCP injection significantly reduced the expression levels of CaMKII, whereas pretreatment with PRO raised them. According to a recent study, long-term memory persistence (LTP) can probably be produced by CaMKII’s involvement in PKMζ formation and autophosphorylation independent of protein synthesis (Sacktor and Fenton, 2018). Furthermore, we discovered that PKMζ expression is higher in the groups treated with medium and high dosages of PRO than in the group treated with SCP. Furthermore, CaMKII promotes CREB phosphorylation using MAPKs. CREB is necessary for neuronal growth, proliferation, differentiation, and survival. Medium and high dosages of PRO showed the greatest protection against SCP-indued models, as evidenced by an increase in CREB levels. In a similar vein, phosphorylated CREB facilitates increased Arc protein translation, which in turn increases BDNF transcription. Pretreatment BDNF expression is higher as compared to SCP and other groups at a higher dose. Additionally, BDNF stimulates the trafficking of AMPA receptors to the cell surface via binding to the receptor TrkB, which is situated on the surface (Guntupalli et al., 2016). Through PRO delivery, this quickens the LTM formation and fortifies synapses through the CREB-BDNF cascade (Figure 8). While these coordinated molecular changes are biologically consistent with enhanced synaptic plasticity, the present findings represent correlative associations and should be interpreted as a putative signaling framework rather than a confirmed causal cascade linking CaMKII, CREB, BDNF, and PKMζ.

A key limitation of the present study is the use of the scopolamine-induced amnesia model, which reflects acute and reversible cholinergic dysfunction rather than the chronic, progressive neuropathology characteristic of AD. Accordingly, the findings should be interpreted as reflecting propranolol’s effects on memory dysfunction, synaptic plasticity, and associated mitochondrial signaling under AD-like amnestic conditions, rather than definitive disease-modifying efficacy in bona fide Alzheimer’s pathology. This model nonetheless remains well suited for probing molecular and behavioral mechanisms underlying cognitive impairment and for generating testable mechanistic hypotheses.

The central actions of propranolol observed in the present study are pharmacologically plausible given its well-established ability to cross the blood–brain barrier and directly modulate β-adrenergic signaling within the brain. Noradrenergic transmission, particularly through β-adrenergic receptors, plays a critical role in regulating memory encoding, emotional arousal, synaptic plasticity, and mitochondrial function. Excessive or dysregulated β-adrenergic activation, such as that occurring during stress or cholinergic imbalance, can impair long-term potentiation, increase oxidative stress, and promote neuroinflammatory responses. By blocking central β-adrenergic receptors, propranolol likely attenuates maladaptive cAMP signaling, thereby stabilizing intracellular calcium dynamics and creating a permissive environment for the activation of plasticity-related pathways including CaMKII, CREB, BDNF, and PKMζ. The observed improvements in mitochondrial membrane potential and reductions in oxidative stress further support the notion that β-adrenergic modulation contributes to improved neuronal metabolic homeostasis under amnestic conditions.

While mitochondrial failure has been linked to various forms of dementia, its precise function in the cognitive impairment brought on by SCP is well documented (Wong-Guerra et al., 2017). After the anti-amnesic activity of PRO was confirmed, we investigated the dynamics of the mitochondria. The characteristic of mitochondrial oxidative stress is reactive oxygen species overproduction, which can cause mutations in the mitochondria’s DNA, damage the respiratory chain, alter membrane permeability, and impact Ca^2+^ homeostasis and the mitochondria’s defensive mechanisms. Our results suggest that mitochondrial damage and oxidative stress may play a part in the development of neurodegenerative diseases in the SCP-induced group. At medium and higher dosages of PRO, there is a considerable reduction in the quantity of ROS in the mitochondria. Similarly, lipid peroxidation is a risk factor for the inner mitochondrial membrane, which is located close to the ROS generation site. The fluidity and other biophysical properties of mitochondria can be altered by the peroxidation of mitochondrial phospholipids, which can also increase the permeability of the inner mitochondrial membrane to protons. The mitochondrial membrane potential is recovered by pretreatment with a medium dosage of PRO, in contrast to the SCP-induced group. All things considered, PRO can provide strategies to modify mitochondrial dysfunction and help in biogenesis (Wills et al., 2012), which may make them attractive therapeutic options for the treatment of many neurodegenerative diseases. However, within the constraints of the SCP model, these mitochondrial improvements are best interpreted as supportive mechanisms underlying memory restoration rather than direct evidence of disease-modifying neuroprotection.

Following PRO’s validation of the recovery of mitochondrial dynamics, we examined the inflammatory response and the removal of Aβ_1–42_ plaque in further detail. All dosages of PRO significantly diminish the Aβ_1–42_ mediated toxicity in the PFC, but the SCP-induced group exhibits Aβ_1–42_ plaque deposition (Dobarro et al., 2013). Neurons in AD release Aβ, which stimulates astrocytes and microglia. More specifically, astrocytes triggered by different cellular stresses have an upregulation of the machinery needed for the formation of Aβ. The injection of SCP caused oxidative stress in the PFC, as evidenced by increased levels of GFAP and TNF-α. Evidence may indicate that reducing TNF-α and GFAP loads with all PRO dosages would therefore contribute to a reduction in AD symptoms (Woiciechowsky et al., 2004). Studies reveal that TNF-α controls the expression of GFAP, the most specific marker for astrocytes, in numerous brain diseases. Moreover, the amyloid cascade theory postulates that Aβ initiates a series of harmful occurrences, including τ hyperphosphorylation and neuroinflammation, that lead to synaptic malfunction and dementia in the end. It could be suggested that the reduction of Aβ burden by PRO would consequently contribute to ameliorating AD symptoms. Within the present experimental context, these effects indicate attenuation of amyloid-associated and inflammatory stress under AD-like conditions, rather than definitive interruption of the amyloid cascade as observed in progressive AD.

In summary, the results of this study demonstrate that PRO exhibits both nootropic and neuroprotective properties when it comes to rat amnesia caused by SCP (Table 1). PRO has anti-amnesic properties that may be mediated by a variety of biochemical pathways, including cholinergic activity, helps in neurogenesis, activates the CREB-BDNF and PKMζ signaling pathways, and CaMKII. Therefore, PRO might be a promising line of treatment for people with neurodegenerative disorders. Despite successful activations in long-term memory restoration during AD pathogenesis, the pretreatment administration of PRO at medium and high doses against SCP-induced wistar rats still falls short of what is needed for effective therapy. Further studies are warranted to improve understanding of the neuroprotective mechanism of PRO and translate the findings to clinical settings. Future investigations employing pathway-specific inhibition, receptor-level dissection, and chronic disease models will be essential to validate causality and determine translational relevance.

**TABLE 1:** Provides a comparative assessment of the molecular markers linked to toxicity and memory in all PFC nootropic and anti-amnesic groups.

Maker’s assessment		DPZ (1 mg/kg)	PRO (10 mg/kg)	PRO (30 mg/kg)	PRO (50 mg/kg)
Cognitive improvement (Nootropic)	Recognition indices %	+		+	+++
	Inflection ratio	++		+	
Cognitive improvement (Anti-amnestic)	Recognition indices %	+	+++	+	+++
	Inflection ratio	+++		+++	
Synthesis of synaptic plasticity markers	CaMKII	+++	+++	+++	+++
	CREB		+++	+++	+++
	BDNF				+++
	PKMζ	+	+	+++	+++
Mitochondrial restoration	Mitochondrial ROS	++		+++	++
	Membrane potential (Δψm)			+	
Inhibition of toxicity, inflammatory markers, and oxidative stress parameter	Aβ deposition	+++	+++	+++	+++
	TNF-α	+++		+++	+++
	GFAP	++	++	+++	+++

## 5 Conclusion

Propranolol administration at specific doses ameliorated SCP-induced cognitive impairment in Wistar rats, as reflected by improved recognition memory, reduced anxiety-like behavior, and preservation of neuronal integrity in the PFC. These behavioral effects were accompanied by coordinated modulation of synaptic plasticity–associated markers, including CaMKII, CREB–BDNF, and PKMζ, alongside restoration of mitochondrial membrane potential, reduction of mitochondrial oxidative stress, and attenuation of inflammatory and amyloid-associated markers such as TNF-α and Aβ. Together, these findings support an association between propranolol treatment and preservation of synaptic and mitochondrial function under AD-like amnestic conditions. However, the study is limited by the use of an acute SCP-induced amnesia model, which reflects reversible cholinergic dysfunction rather than progressive AD pathology, and by the associative nature of the molecular findings, which do not establish causal signaling relationships. Nevertheless, this work provides an integrated behavioral-to-molecular mechanistic framework that generates testable hypotheses for future studies employing pathway-specific interventions, chronic neurodegeneration models, and translational approaches to explore the therapeutic relevance of PRO in cognitive and neurodegenerative disorders.

## Data Availability

The raw data supporting the conclusions of this article will be made available by the authors, without undue reservation.
