# Supplementary figures and images for: Propranolol reinstates mitochondrial dynamics and synaptic memory pathways through CaMKII/CREB–BDNF/ PKMζ cascades in an AD-like rat model
Source: Front Aging Neurosci. 2026 May 29;18:1729046. doi: 10.3389/fnagi.2026.1729046 (PMC13261808; doi:10.3389/fnagi.2026.1729046)

Full blots

**BDNF (28 kDa)**


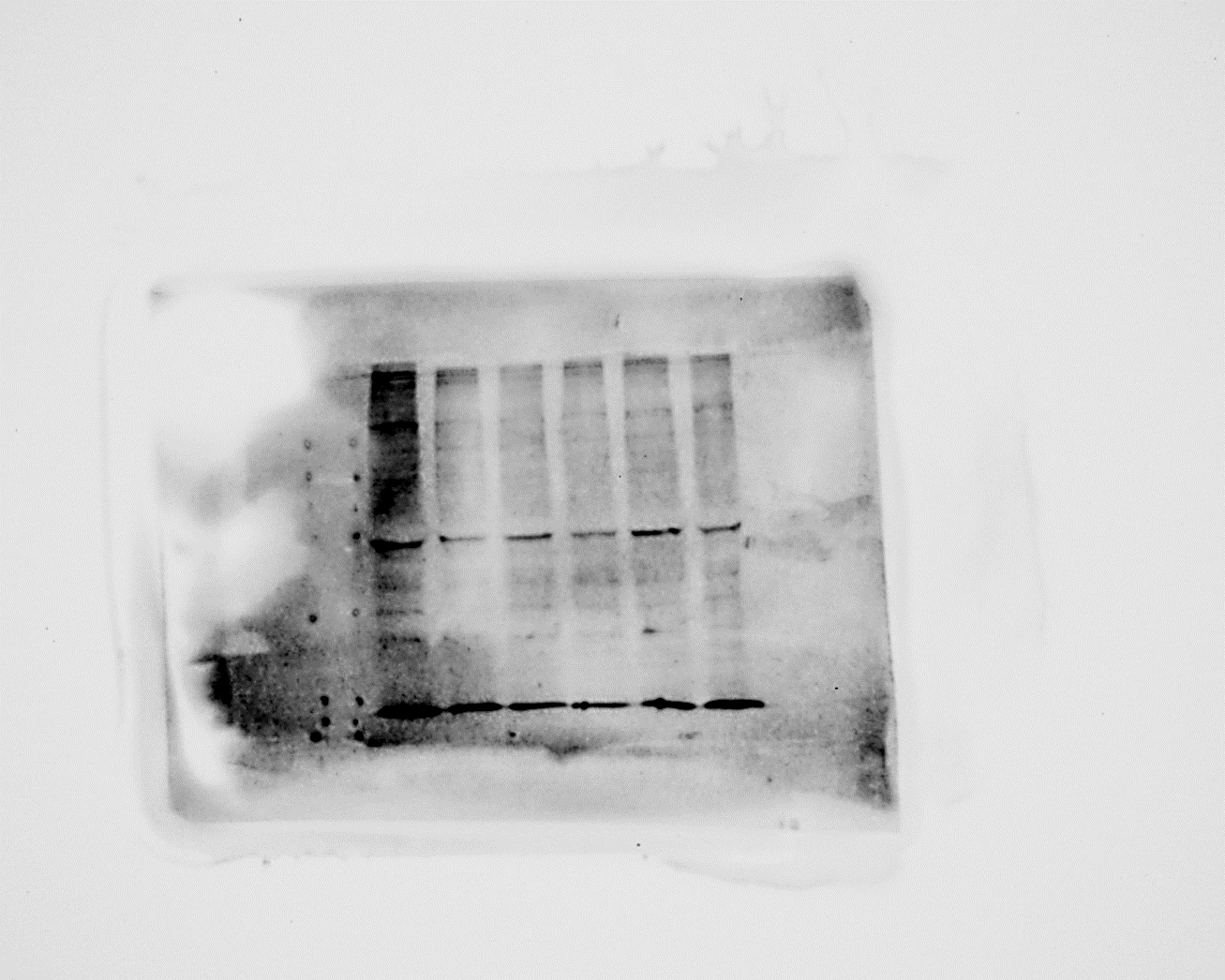


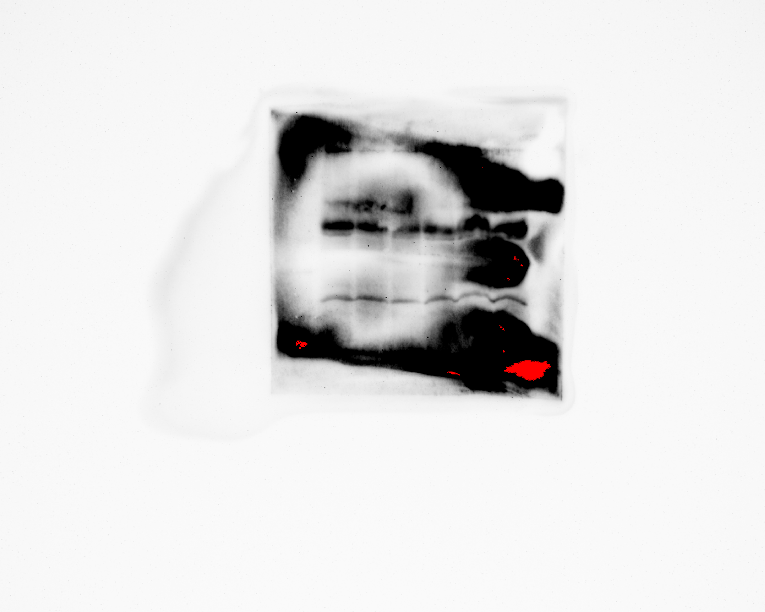


**PKM ζ (78 kDa)**

**GAPDH (36 kDa)**


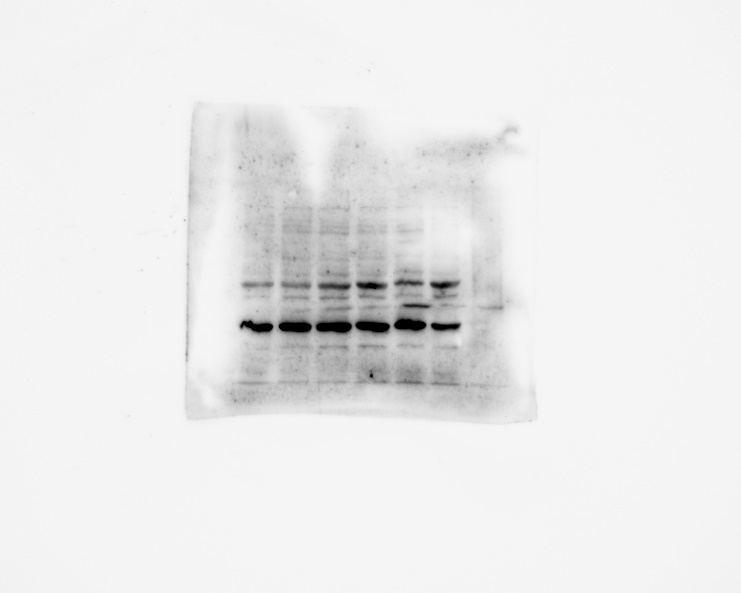

Supplement: Supplementary file 1 [file Data_Sheet_1.docx]
